# Acute Exposure to Microcystin-Producing Cyanobacterium *Microcystis aeruginosa* Alters Adult Zebrafish (*Danio rerio*) Swimming Performance Parameters

**DOI:** 10.1155/2011/280304

**Published:** 2011-12-28

**Authors:** Luiza Wilges Kist, Angelo Luis Piato, João Gabriel Santos da Rosa, Gessi Koakoski, Leonardo José Gil Barcellos, João Sarkis Yunes, Carla Denise Bonan, Maurício Reis Bogo

**Affiliations:** ^1^Laboratório de Biologia Genômica e Molecular, Faculdade de Biociências, Pontifícia Universidade Católica do Rio Grande do Sul, Avenida Ipiranga 6681, 90619-900 Porto Alegre, RS, Brazil; ^2^Instituto Nacional de Ciência e Tecnologia Translacional em Medicina (INCT-TM), Hospital de Clínicas, Rua Ramiro Barcelos 2350, 90035-003 Porto Alegre, RS, Brazil; ^3^Laboratório de Neuroquímica e Psicofarmacologia, Faculdade de Biociências, Pontifícia Universidade Católica do Rio Grande do Sul, Avenida Ipiranga 6681, 90619-900 Porto Alegre, RS, Brazil; ^4^Programa de Pós-Graduação em Farmacologia, Centro de Ciências da Saúde, Universidade Federal de Santa Maria, Avenida Roraima 1000, 97105-900 Santa Maria, RS, Brazil; ^5^Curso de Medicina Veterinária, Universidade de Passo Fundo, Campus Universitário, Caixa Postal 611, 99001-970 Passo Fundo, RS, Brazil; ^6^Unidade de Pesquisas em Cianobactérias, Prédio da Hidroquímica, Instituto de Oceanografia, Campus Carreiros da FURG, Caixa Postal 474, Rio Grande, RS, Brazil

## Abstract

Microcystins (MCs) are toxins produced by cyanobacteria (blue-green algae), primarily *Microcystis aeruginosa*, forming water blooms worldwide. When an organism is exposed to environmental perturbations, alterations in normal behavioral patterns occur. Behavioral repertoire represents the consequence of a diversity of physiological and biochemical alterations. In this study, we assessed behavioral patterns and whole-body cortisol levels of adult zebrafish (*Danio rerio*) exposed to cell culture of the microcystin-producing cyanobacterium *M. aeruginosa* (MC-LR, strain RST9501). MC-LR exposure (100 **μ**g/L) decreased by 63% the distance traveled and increased threefold the immobility time when compared to the control group. Interestingly, no significant alterations in the number of line crossings were found at the same MC-LR concentration and time of exposure. When animals were exposed to 50 and 100 **μ**g/L, MC-LR promoted a significant increase (around 93%) in the time spent in the bottom portion of the tank, suggesting an anxiogenic effect. The results also showed that none of the MC-LR concentrations tested promoted significant alterations in absolute turn angle, path efficiency, social behavior, or whole-body cortisol level. These findings indicate that behavior is susceptible to MC-LR exposure and provide evidence for a better understanding of the ecological consequences of toxic algal blooms.

## 1. Introduction

*Microcystis aeruginosa* is a freshwater cyanobacteria, known producer of a family of toxins termed microcystins (MCs) [1, 2]. MCs are hepatotoxic cyclic heptapeptides released into water during or on senescence of cyanobacterial blooms [3]. The peptide rings of MCs contain five nonprotein amino acids, whereas the two-protein amino acids distinguish MCs from one another. MC-LR contains the amino acids leucine and arginine. MC-LR is one of the most commonly occurring [2, 4] and the most toxic microcystin [5]. The intact cells as well as the toxins released after cellular lysis can be responsible for the toxic effects observed in many organisms, from microalgae [6] to mammals [7] including human [8–10].

Exposure to toxic cyanobacteria or administration of MCs may cause hepatotoxic effects [11–13], oxidative stress [14], kidney damage [15, 16], growth inhibition [17, 18], reproductive injury [19], haematological and biochemical alterations [20–22], apoptosis [23], and even fish death [24].

Alterations in normal behavioral patterns may be the first line of defense when an animal is exposed to an environmental perturbation [25–28]. Additionally, studies have shown important interrelationships between hormones and behavior [29–33]. Thus, alteration in cortisol level may consequently alter normal fish behavior. The effects of MC on fish behavior are still unknown, but some issues have already been addressed. Baganz et al. [34, 35] reported changes in the spontaneous locomotor behavior of zebrafish (*Danio rerio*) and *Leucaspius delineatus* after MC-LR exposure, and Cazenave et al. [36] showed changes in swimming activity of *Jenynsia multidentata* fed with microcystin-RR (MC-RR). In addition, studies using different exposure routes (intraperitoneal injection, oral ingestion, or immersion) have demonstrated that MCs can accumulate in fish tissues, mainly in the liver [21, 36–39], intestine [37, 39–41], gills [42, 43], kidney [37, 39], muscle [40, 41, 44–46], gallbladder [47], blood [40, 41, 48], and brain [43]. Altogether, these findings indicate possible neurotoxic effects of MCs on fish, causing serious risks to the success of fish populations and changes in biodiversity, among other ecological consequences [36].

The zebrafish is rapidly becoming a popular model species in many areas of biological research. Its application includes the fields of developmental biology [49], toxicology [50], neurophysiology, biomedicine, drug discovery [51], human diseases [52–54], pharmacology and behavioral analysis [55–59]. These fish exhibit robust behavioral responses, well-characterized genome, neural and endocrine systems homologous to humans [60–62], and possess all of the “classical” vertebrate neurotransmitters [63, 64]. Additionally, zebrafish are an ideal animal model for laboratory research because they are inexpensive, require low maintenance, and produce abundant offspring [65]. Recently, this fish was also used for proteomic studies on the toxicity of MCs [66, 67].

In order to better understand the neurotoxic effects of MCs on fish and to improve the knowledge of mechanisms underlying the toxicity, the main goal of this study was to assess the effects of MC-LR on zebrafish behavioral parameters and endocrine (whole-body cortisol) response after toxin exposure.

## 2. Materials and Methods

### 2.1. Animals

Wild-type adult (<8 months old) zebrafish (*Danio rerio*) of both sexes were obtained from specialized supplier (Redfish Agroloja, RS, Brazil). Animals were kept in 50 L housing tanks with tap water previously treated with Tetra's AquaSafe (to neutralize chlorine, chloramines, and heavy metals present in the water that could be harmful to fish) and continuously aerated (7.20 mg O_2_/L) at 26 ± 2°C, under a 14–10 h light/dark photoperiod in a density of up to five animals per liter. Animals were acclimated for at least two weeks before the experiments. They were fed three times a day with TetraMin Tropical Flake fish.

 The procedures were previously approved by the Animal Ethics Committee of Pontifical Catholic University of Rio Grande do Sul (PUCRS) under the protocol number 10/00142-CEUA.

### 2.2. Treatments

The amount of MC-LR in the cell culture of *M. aeroginosa* (strain RST9501) was detected by a Quantitative Antibody Immunoassay (Elisa) against MC-LR provided by Envirologix (Portland, USA), within a range of detection from 0.05 to 2.5 *μ*g/L MCs. A suitable dilution was applied to the culture sample to provide detection within the range. Zebrafish were distributed in three groups: the first group (controls) was exposed to water containing the culture medium of *M. aeroginosa* for 24 hours; the second and third groups were exposed to cell culture in a final MC-LR concentration of 50 *μ*g/L and 100 *μ*g/L during 24 hours, respectively. Immediately after the exposure, animals were tested in tank-diving behavioral test and social interaction. After behavioral tests animals were euthanized by decapitation.

The MC-LR concentrations and the time of exposure were chosen based on a previous study using *J. multidentata* [43]. Besides, such concentrations are commonly encountered in cyanobacterial bloom events [68, 69].

### 2.3. Tank-Diving Behavioral Test

Behavioral testing took place during the light phase between 10:00 AM and 4:00 PM The animals were individually transferred to a 2.7 L tank (24 cm L × 8 cm W × 20 cm H) with laterals and bottom white covered to avoid any visual disturbances and habituated to the tank for 30 s, as previously described [70]. There was no drug exposure during behavioral experiments. The locomotor activity of the animals was video-recorded using Logitech Quickcam PRO9000 for five minutes after the habituation period and further analyzed using the ANY-Maze recording software (Stoelting Co., Wood Dale, IL, USA). The tank was divided into equal sections with four vertical lines and one horizontal line, and the following behavior patterns were measured: distance traveled (meters), immobility time (seconds), number of crossings, absolute turn angle, path efficiency, and time (seconds) spent in the bottom portion. This task exploits the natural tendency for zebrafish to spend most of the time at the bottom when introduced into a novel environment and then gradually extend the swimming range, over a period of minutes, to include the upper portions of the test tank. A longer time spent in the bottom part of the tank indicates heightened anxiety [71].

### 2.4. Social Interaction

Zebrafish is a schooling fish that may exhibit preference for its conspecifics under certain circumstances. The social interaction analysis was based on Gerlai [72]. After 24 hours of exposition to 50 or 100 *μ*g/L of MC-LR, fish were placed in groups of five in a small experimental tank (30 cm L × 15 cm H × 10 cm W). On one side of the experimental tank, an empty fish tank was placed, and, on the other side, there was a tank of identical size containing 15 conspecifics. The experimental fish were allowed to acclimate to the experimental tank for a 30 s period, after which their behavior was analyzed. The next 10 s of this test was analyzed as follows. The experimental tank was virtually divided into two equal sections with one vertical line. The time that all five experimental fish spent on the side of the tank closer to the conspecific school was measured using a stopwatch.

### 2.5. Acute Restraint Stress (ARS) Protocol

The ARS protocol was based on Piato et al. [73]. Following the habituation period, fish were submitted to the ARS protocol. This experiment consisted in keeping each animal enclosed into microcentrifuge plastic tubes of 2 mL with the cap closed and small openings in both ends to allow free water circulation inside the tube and completely avoid fish locomotion. After 90 min of confinement, animals were gently captured and immediately frozen in liquid nitrogen and stored at −80°C until cortisol extraction. Aeration (8 ppm, Labcom Test Camboriú, SC, Brazil) and water temperature (26 ± 2°C) were controlled throughout the test.

### 2.6. Measurement of Cortisol

The extraction and measurement of whole-body cortisol from zebrafish have been described in detail by Barcellos et al. [74]. Briefly, zebrafish were distributed in four groups: the first group, which consisted of zebrafish exposed to water containing the culture medium of *M. aeroginosa* for 24 hours, was considered the “negative control”; the second and third groups were exposed to cell culture in a final MC-LR concentration of 50 *μ*g/L and 100 *μ*g/L during 24 hours, respectively; in the fourth group, considered the “positive control,” zebrafish were submitted to the ARS protocol. After, zebrafish were captured and immediately frozen in liquid nitrogen and stored at −80°C until whole-body cortisol extraction. Each zebrafish was weighed, and a pool of three fish was minced and placed into a disposable stomacher bag with 2 mL of phosphate buffered saline (PBS, pH 7.4) for 6 min. The contents were transferred to a 10 mL screw top disposable test tube, and 5 mL of laboratory grade ethyl ether was added. The tube was vortexed for 1 min and centrifuged for 10 min at 3000 rpm. The tube was then immediately frozen at liquid nitrogen, and the unfrozen portion (ethyl ether containing cortisol) was decanted. The ethyl ether was transferred to a new tube and completely evaporated under a gentle stream of nitrogen for 2 h, yielding a lipid extract containing the cortisol. The extract was stored at −20°C until the ELISA was conducted on the samples suspended with 1 mL of PBS buffer. In order to prevent a possible stress response induced by manipulation, the time elapsed between capture and killing was less than 10 s. Whole-body cortisol was measured in duplicate samples of tissue extract with a commercially available high sensitivity salivary cortisol-enzyme immunoassay kit (Salimetrics, USA). The specificity of the test was evaluated by comparing the parallelism between the standard curve and serial dilutions of the tissue extracts in PBS (pH 7.4). The standard curve constructed with the human standards ran parallel to that obtained using serial dilutions of zebrafish tissue extracts. In the linear regression test, high positive correlation (*R* ^2^ = 0.9818) was found between the curves. The intra-assay coefficient of variation was 3.33–3.65%.

### 2.7. Statistical Analysis

Data of the exploratory assessment, social interaction, and cortisol levels were expressed as mean ± SEM and analyzed by one-way ANOVA, followed by Newman-Keuls post hoc test. A significant difference was attributed to *P* < 0.05. All data were evaluated by SPSS 18.0 for Windows.

## 3. Results

**Figure 1:**
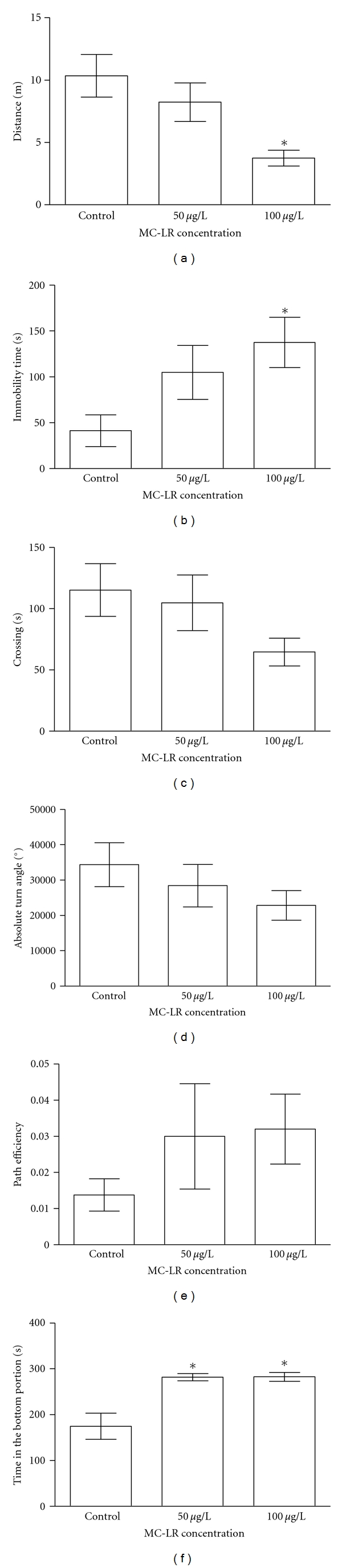
Effect of microcystin-LR exposure on the distance traveled (a), immobility time (b), number of line crossings (c), absolute turn angle (d), path efficiency (e), and time in the bottom portion (f) determined during 5 min of video recording in the tank-diving behavioral test. Data expressed as mean ± SEM. *n* = 10. One-way ANOVA/Newman-Keuls post hoc test. *: *P* < 0.05 compared to control group.

Distinct parameters of zebrafish swimming activity were evaluated in the 5-min tank-diving behavioral test. MC-LR exposure at 100 *μ*g/L significantly (one-way ANOVA/Newman-Keuls, *P* < 0.0081, *n* = 10) decreased the distance traveled (3.7 ± 0.6 meters) in relation to control animals (10.3 ± 1.7 meters) (Figure 1(a)) and significantly (one-way ANOVA/Newman-Keuls, *P* < 0.039, *n* = 10) increased the immobility time (137.6 ± 27.6 seconds) when compared to the control group (41.5 ± 17.3 seconds) (Figure 1(b)) whereas MC-LR exposure at 50 *μ*g/L did not alter both parameters. No differences in the number of line crossings, absolute turn angle, and path efficiency were observed in both concentrations tested (Figure 1(c), 1D and 1E, resp.).

Control animals spent 58.4% of time (175.0 ± 28.6) in the bottom portion of the test tank. Animals exposed to 50 and 100 *μ*g/L MC-LR significantly (one-way ANOVA/Newman-Keuls, *P* < 0.0003, *n* = 10) increased (93%) the time spent in the bottom portion of the test tank (282.1 ± 7.90 and 282.7 ± 9.7, resp.) when compared with control group (175.0 ± 28.6) (Figure 1(f)).

**Figure 2:**
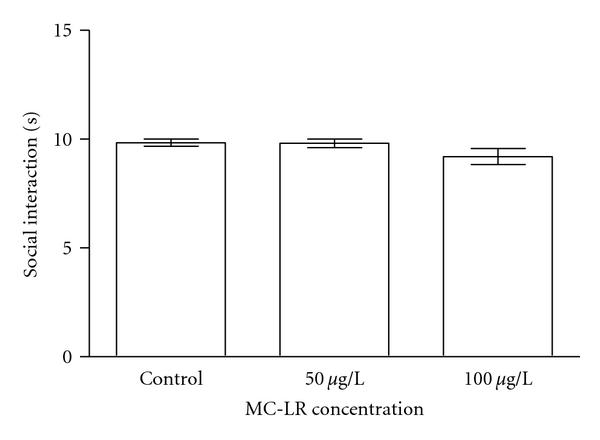
Effect of exposure to microcystin-LR on social interaction. Data expressed as mean ± SEM. *n* = 10. One-way ANOVA/Newman-Keuls post hoc test.

In relation to social interaction test, the results showed that 50 and 100 *μ*g/L of MC-LR concentrations did not promote any alteration in the animals regarding this behavior (Figure 2).

**Figure 3:**
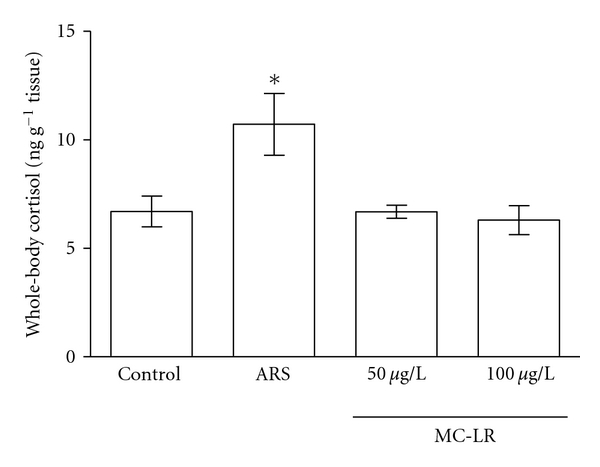
Effect of exposure to microcystin-LR on whole-body cortisol levels. Data expressed as men ± SEM. *n* = 7. One-way ANOVA/Newman-Keuls post hoc test. *P < 0.05 compared to control group.

 Levels of whole-body cortisol also were measured. The ARS protocol (positive control) resulted in enhanced whole-body cortisol in relation to control group (one-way ANOVA/Newman-Keuls, *P* < 0.005, *n* = 7; 10.7 ± 1.4 and 6.7 ± 0.7, resp.). Zebrafish treated with both concentrations of MC-LR did not present altered levels of cortisol in relation to control group (Figure 3).

## 4. Discussion

Behavioral alterations reflect how an animal senses and responds to its environment and is the first line of defense when an animal is exposed to an environmental perturbation [28]. Since it was already demonstrated that the effects promoted by cyanobacterial crude extracts on aquatic organisms were either more pronounced or different from those observed using pure toxins [75, 76], we used cell culture of the microcystin-producing cyanobacterium *M. aeruginosa* (MC-LR) in order to evaluate the effects of MCs on zebrafish behavior.

The toxin concentration and time period of animals' exposure were chosen based on previous studies that showed MCs accumulation in fish tissues [21, 37–48]. The results presented herein demonstrated that 100 *μ*g/L MC-LR decreased the distance traveled and increased the immobility time. However, no significant alterations were found in the number of line crossings with both concentrations. When animals were exposed to 50 and 100 *μ*g/L, MC-LR led to a significant increase in the time spent in the bottom portion. The results also showed that none of the MC-LR concentrations tested promoted significant alterations in the absolute turn angle, path efficiency, or social interaction.

Since behavior links physiological function with ecological processes for a given species, it might provide a useful indicator or biomarker for detecting harmful chemical pollutants [77]. The potential of the zebrafish as a model in neurobehavioral research has emerged only recently. Studies have examined behavior in zebrafish larvae [78–81], as well as their responses to different drugs, such as ethanol [82, 83] and fluoxetine [84]. Studies on adult zebrafish include social behavior [85–87], olfactory-related behaviors [88, 89], anxiety [74], addiction [90–92], sleep [93], learning and memory [94, 95].

There are still only few studies evaluating the effects of MCs on fish behavior. Baganz et al. [34] verified that exposure to MC-LR caused dose-effect-related changes in spontaneous locomotor activity in zebrafish. Whereas exposure to lower concentrations (0.5 and 5 *μ*g/L) caused an increase in daytime mobility, elevated exposures (15 and 50 *μ*g/L) led to significantly increased immobility. The highest exposure (50 *μ*g/L) also reduced the spawning activity and reduced spawning success. In contrast to daytime activities, night-time swimming activity was significantly greater at the higher MC-LR exposures. In another study, Baganz et al. [35] showed changes in the spontaneous locomotor behavior of zebrafish and *L. delineatus* after exposure to MC-LR in concentrations of 0.5, 5, and 15 *μ*g/L for 17 days and 50 *μ*g/L for six days. During the daytime, the mobility of zebrafish as well as *L. delineatus* increased significantly by exposure to the lowest concentrations, whereas higher concentrations led to significantly decreased mobility. Influenced by MC-LR, the swimming time of *L. delineatus* reversed, going from a prominently diurnal activity to a nocturnal one; zebrafish remained active during the daytime. Additionally, Cazenave et al. [36] reported changes in the swimming activity of *J. multidentata* fed with contaminated food pellets containing MC-RR. Low levels (0.01 *μ*g/g) increased the swimming activity, while the highest dose (1 *μ*g/g) used produced significant changes with respect to control group (only since approximately 20 hours of exposure), when the swimming activity was decreased.

In this sense, our findings demonstrate that MC-LR at the highest concentration (100 *μ*g/L) caused a decrease in the distance traveled and an increase in the immobility time in zebrafish. Interestingly, no significant alterations in the number of line crossings were found at the same MC-LR concentration and time of exposure, despite the tendency to decrease the number of crossings in greater concentration. It is important to emphasize that these findings are similar to the results published previously by Baganz et al. [35]. However, these authors have used purified toxin whereas a cell culture of the microcystin-producing cyanobacterium *M. aeruginosa* (MC-LR) was used in our experiments. Reduction in swimming capability, resulting in a reduction in the rate of activity, may decrease the ability to gather food and make the fish more vulnerable to predation [96]. Under natural conditions, this reduced overall level of activity will eventually cause disadvantages to the organisms in the ecosystem, and, therefore, influence the biocoenotic structures and functions [34].

Exposure to a novel environment evokes a robust anxiety response in zebrafish [97], as they dive to the bottom (geotaxis) until they feel safe to swim in the upper regions of the tank [58]. Here, MC-LR at 50 and 100 *μ*g/L promoted an increase in the time spent in the bottom portion, suggesting an anxiety behavior.

The zebrafish is a social species and exhibits group preference as well as aggression. Shoaling behavior commences soon after hatching and fish reared in isolation quickly form shoals when placed together [98]. One study has demonstrated that exposure to nonylphenol over a 5-day period decreased shoaling tendency in juvenile rainbow trout (*Oncorhynchus mykiss*) [99]. Similarly, herbicide-exposed goldfish also showed a decreased aggregation [100]. Locomotor activity, aggressive behavior, and group preference of the male zebrafish and group preference of the females were clearly inhibited when zebrafish were exposed for 60 days to 100 *μ*g/L nonylphenol concentration [101]. For this reason, we evaluated the effect of MC-LR exposure on zebrafish social interaction. However, no significant alteration was found between control and MC-LR-exposed animals.

Studies have shown important interrelationships between stress hormones and behavior [29–33]. An elevated plasma cortisol level is a primary indicator of a stress response in fish [102]. Zebrafish, like humans, employ cortisol as a primary stress response hormone [74]. Considering this, we measured whole-body cortisol in zebrafish to verify if MC could elicit a stress response in treated fish. However, no significant alterations were found in whole-body cortisol levels in animals exposed to both concentrations of MC-LR compared to the control group. Barcellos et al. [74] demonstrated that whole-body cortisol level of zebrafish increases after visual contact with a predator species. Cortisol levels were significantly higher in zebrafish submitted to unpredictable chronic stress (UCS) protocol when compared to control group [103]. Bury et al. [104] reported a significant increase in plasma cortisol levels of the brown trout (*Salmo trutta*) after 1 h and returned to the control level after 24 h of exposure to lysed toxic *Microcystis aeruginosa* cells. Crucian carp (*Carassius auratus*) exposed to sublethal and lethal doses (150 *μ*g/kg and 600 *μ*g/kg, resp.) of *Microcystis* extracts exhibited a significant acute increase in plasma cortisol levels, which suggested that MC elicited a stress response in treated fish. The profiles of cortisol changes in fish treated with MC appeared to be dose dependent, indicating that fish in the high-dose group experienced greater MC-induced disturbance [105].

In summary, behavioral response of fish may be a promising biomarker of sublethal toxicity and water contamination. Several behavioral endpoint measurements, especially locomotor activity and the time spent in the bottom portion, may provide an effective assessment of MCs in aquatic ecosystem.
